# Understanding Metabolic Alterations in Cancer Cachexia through the Lens of Exercise Physiology

**DOI:** 10.3390/cells11152317

**Published:** 2022-07-27

**Authors:** Irina Kareva

**Affiliations:** Department of Biomedical Engineering, Northeastern University, Boston, MA 02115, USA; irina.kareva@alumni.harvard.edu

**Keywords:** cachexia, cancer, metabolism, metabolic derangement, ventilatory thresholds, metabolic flexibility

## Abstract

Cancer cachexia is one of the leading causes of mortality for late-stage cancer patients. One of its key characteristics is abnormal metabolism and loss of metabolic flexibility, i.e., loss of ability to switch between use of fats and carbohydrates as needed. Here, it is hypothesized that late-stage systemic cancer creates a chronic resource drain on the body that may result in the same metabolic adaptations that occur during intense endurance exercise, activating some of the same mechanisms of nutrient consumption that are supposed to be transient during strenuous physical activity. This hypothesis is evaluated by creating a mathematical model that characterizes the relationships between increased exercise intensity and carbohydrate and fat oxidation. The model is parametrized using published data on these characteristics for a group of professional athletes, moderately active individuals, and individuals with metabolic syndrome. Transitions between different zones of relative nutrient consumption as a function of increased effort are captured through explicitly modeling ventilatory thresholds, particularly VT1 and VT2, where fat is primarily used below VT1, both carbohydrates and fats are used between VT1 and VT2, and where carbohydrates become the primary source of fuel above VT2. A simulation is conducted of projected patterns of nutrient consumption when simulated “effort” remains between VT1 and VT2, or above VT2, and it is proposed that it is the scenario when the simulated effort is maintained primarily above VT2 that most closely resembles metabolic patterns characteristic of cachexia. A discussion of a broader framework for understanding cachectic metabolism using insights from exercise physiology, including potential intervention strategies, concludes this paper.

## 1. Introduction

Cancer cachexia is a systemic irreversible multi-organ syndrome characterized by elevated protein breakdown, inflammation, loss of skeletal muscle and adipose tissue, and metabolic derangement [1,2,3]. It occurs in over half of late-stage cancer patients, with the highest incidence in patients with gastric and pancreatic cancer [1]. One of the striking aspects of cancer cachexia is abnormal metabolism, c alterations in preferential nutrient consumption and lack of metabolic flexibility, i.e., the ability to utilize both carbohydrates and fats, and switch between them as needed [4].

Interestingly, preferential nutrient consumption has been studied extensively in the area of exercise physiology. Relative nutrient depletion as a function of effort is typically measured using an indirect calorimeter [5,6]. Individuals wear a tightly fitted mask over their nose and mouth that measures the amount of oxygen (ventilatory oxygen, or VO_2_, typically measured in L/min) consumed and the amount of carbon dioxide (VCO_2_, typically measured in L/min) exhaled. While wearing the mask, individuals exercise on an ergometer, such as a treadmill or an exercise bike, incrementally increasing effort through increasing speed or resistance. Changes in VO_2_ and VCO_2_ as a function of exercise intensity can then be calculated at each time point according to equations reported by Frayn [7]. These are the data that were collected and reported by San Millan and Brooks [8] for professional athletes (PA), moderately active individuals (MA) and individuals with metabolic syndrome (MtS). Individuals with metabolic syndrome, who are diagnosed as having three out of five criteria, including large waist, high level of triglycerides, reduced HDL cholesterol, increased blood pressure and elevated fasting blood sugar, are more likely to develop a cluster of co-occurring conditions, including heart disease, stroke, type 2 diabetes [9], and have an increased risk of cancer [10].

In [8], San Millan and Brooks recorded change in CHOox and FATox as a function of power output (W), showing differences in metabolism in individuals with different levels of fitness. Specifically, they show that for all participants, as wattage increases, fat oxidation decreases, while carbohydrate metabolism increases, until at high power output fat oxidation tends to zero. While the shape of the reported power-metabolism curves remains similar for the three groups of participants, qualitatively they become left-shifted as the level of fitness decreases, such that transitions between types of nutrients that are being used occur at lower exercise intensity for less fit individuals. Notably, in the reported data, for professional athletes the fat oxidation curve first increases as a function of workload and then decreases; this is most likely an artifact of the protocol used: gas exchange outputs were recorded for all individuals starting at the absolute workload of 100 Watts, which is likely to already be quite high intensity for all but professional athletes. If the data were recorded starting at lower workload levels, the reported curves would most likely mirror those for professional athletes but would be significantly left-shifted.

One approach to understand the points at which these metabolic switches occur, i.e., when an individual transitions from using fat as a primary fuel source to using carbohydrates, is through the lens of ventilatory thresholds VT1, VT2 and VO_2_max [11,12,13,14]. As exercise intensity increases, first ventilatory threshold (VT1) can be identified as a point when breathing rate starts to increase but it is still possible to carry on a conversation; at the second ventilatory threshold (VT2), the intensity is high enough that fatty acid oxidation is no longer sufficient to meet the demand for energy, resulting in upregulation of carbohydrate metabolism, both aerobic and then anaerobic. This results in increased levels of measurable blood lactate as it can no longer be contained in the muscle and is released into the blood [15]. Typical level of blood lactate associated with this transition is around 2 mmol/L; transition to exercise beyond this threshold can be observed as a point where lactate levels start increasing dramatically (see Figure 1 in [8]). Finally, as exercise intensity increases further, one reaches a limit in how much oxygen the cells are capable of using, where an incremental increase in exercise intensity no longer results in increased oxygen consumption. This point is referred to as VO_2_max, and while technically it is possible to continue exercising beyond this point, physiologically it becomes so difficult that it is unsustainable.

**Figure 1:**
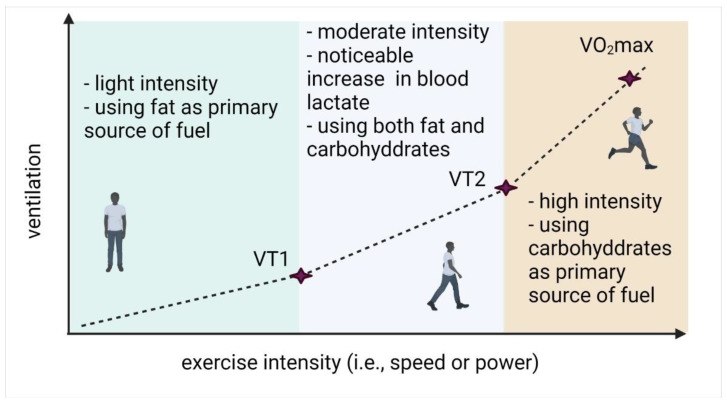
Schematic representation of the relationship between ventilation and exercise intensity as a function of ventilatory thresholds.

A summary of ventilatory thresholds and corresponding changes in metabolism as a function of exercise intensity are given in Figure 1.

In their work, San Millan and Brooks [8] showed that, for individuals with metabolic syndrome, the level of intensity necessary to transition from using fat as a primary fuel source to using carbohydrates is very low. Indeed, it has been shown that respiratory exchange ratio (RER), defined as a ratio between metabolic production of CO_2_ and O_2_ uptake, is below 1 (typically around 0.8) when fat is used as a primary source of fuel, and is close to 1 during intense exercise, or for individuals with metabolic syndrome [16]. That is, for a patient with metabolic syndrome, an incremental increase in activity may result in qualitative transition from using fat as a primary fuel source to carbohydrates. Within the framework of ventilatory thresholds, this would mean that their baseline energy expenditure is near the VT1 threshold.

Given that in patients with cachexia relative energy stores of carbohydrates become the primary source of fuel and thus are depleted first, followed by fat and then protein [17,18], it is hypothesized here that metabolic derangement observed in cachexia can be evaluated within the framework of metabolic thresholds. Specifically, it is hypothesized that the drain on the body’s resources caused by systemic tumor burden may mimic metabolic adaptations of high intensity exercise. 

To test this hypothesis, a phenomenological mathematical model is proposed that captures relationships between exercise intensity and fat and carbohydrate metabolism reported by San Millan and Brooks [8]. A regimen is then simulated where effort gets “stuck” between different thresholds and projected nutrient consumption patterns are observed. These projections are then compared against available nutrient consumption rates reported in the literature, suggesting that metabolic alterations in cachexia could indeed be viewed through within this framework. A discussion of next steps concludes this work, including a description of a sample protocol for validation of the proposed hypothesis in patients, as well as possible interventions.

## 2. Materials and Methods

The proposed phenomenological model describes the following mechanisms. Effort is introduced extrinsically corresponding to the exercise protocol described in [8], where measurements were recorded after participants warmed up for 15 min at an intensity below 100 W. After the warm-up period, participants exercised on a stationary bike, with exercise intensity increasing by 35 W every 10 min, until volitional exhaustion.

To simulate this level of intensity, Effort is put into the model starting at 100 W at 15 min, increasing incrementally to match the reported exercise protocol. The model then describes increase in VO_2_ and VCO_2_ as a function of Effort, such that rates of respiratory gas increase are different when Effort is below VT1, when it is between VT1 and VT2 and when it is above VT2. A full description and derivation of the model is given in Supplementary Materials.

**Figure 2:**
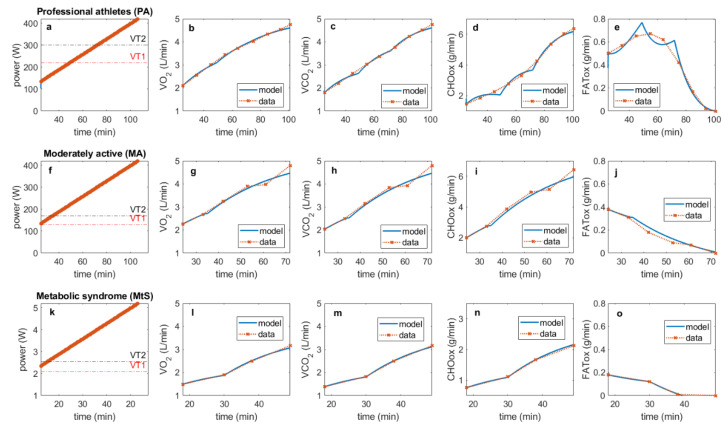
Simulation of experimentally observed relationship between exercise intensity for professional athletes (top row), moderately active individuals (middle row), and individuals with metabolic syndrome (bottom row). (**a**,**f**,**k**) A simulation of exercise, increasing by 35W every ten minutes starting from time t =10 min, with simulations in figures reported from time t = 25 min due to the format of collected data for PA, MA and MtS respectively; (**b**,**g**,**l**) calculated VO_2_ for PA, MA and MtS respectively; (**c**,**h**,**m**) calculated VCO_2_ for PA, MA and MtS respectively; (**d**,**i**,**n**) oxidized carbohydrates CHOox for PA, MA and MtS respectively; (**e**,**j**,**o**) oxidized fatty acids FATox for PA, MA and MtS respectively.

The data for this analysis were obtained from the aforementioned publication by San Millan and Brooks, as reported in Tables 2–4 of [8]. In the current analysis, the specific focus is on reproducing the experimentally observed patterns of fat and carbohydrate oxidation in the three studied groups. The fit of this model to the reported data is shown in Figure 2 (a–e for professional athletes, f–j for moderately active individuals and k–o for individuals with metabolic syndrome). Parameters used to reproduce data in Figure 2 are given in the Supplementary Materials.

### Simulation of “Tumor-like” Effort

Next, the model is used to simulate a situation when Effort either remains between VT1 and VT2, or above VT2. It is hypothesized that due to the systemic drain of metastatic tumor burden on the body, one of these scenarios may be consistent with patterns of nutrient consumption in cachectic patients.

**Figure 3:**
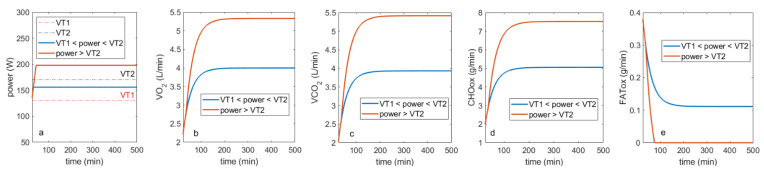
Simulation of projected patterns of relative nutrient consumption for when (**a**) simulated effort persists between VT1 and VT2 (blue), or is above VT2 (red), and corresponding (**b**) projected VO_2_, (**c**) VCO_2_, and (**d**) calculated levels of carbohydrate and (**e**) fatty acid oxidation.

In Figure 3, both of these scenarios are simulated using fits for moderately active individuals as a test case. Effort initiation is simulated as in the previous case, but after Effort reaches a level between either VT1 and VT2 (blue lines), or over VT2 (red lines), it becomes fixed. As can be seen in Figure 3, when Effort is between VT1 and VT2 (Figure 3a), both fats and carbohydrates continue to be oxidized (Figure 3b,c); however, when Effort is above VT2, fat utilization tends to zero, and carbohydrates become the primary nutrient used.

At least in mouse models of cachexia, it appears that carbohydrate stores become depleted first, followed by fat and then protein [17,18]. Therefore, I propose that a scenario, where Effort > VT2 is the one that corresponds more closely to describing cachectic metabolism within the frameworks of the proposed model. Specifically, when effort > VT2, it is carbohydrate metabolism that is preferred (Figure 3d) compared to fatty acid oxidation (Figure 3e).

## 3. Discussion

Most if not all adaptations that are observed in cancer progression have normal equivalents, which are either engaged by cancer in the wrong context (i.e., cell migration), or start normally but become pathological (i.e., wound healing becoming angiogenesis [19,20,21], or transient immune suppression during wound healing or pregnancy [22,23]). While starvation is a natural suspect for such an equivalent to cachexia, the metabolic alterations occurring in starvation are qualitatively different [17,24,25]. Here I hypothesize that, instead, such an equivalent may lie in metabolic adaptations that occur during intense exercise, which evolved to be transient, but in cancer can become more permanent. Specifically, given that at high levels of exercise intensity qualitative changes in relative consumption of carbohydrates relative to fats are observed, I hypothesize that metabolic demands of metastatic tumor burden may initiate the same adaptations, effectively “mimicking” ongoing high effort activity even without such external effort. That is, it is hypothesized that late-stage systemic cancer creates such a resource drain on the body that it activates the same mechanisms of nutrient consumption that are supposed to be transient during exercise.

To evaluate possible implications of this hypothesis, a model is proposed that describes phenomenologically the patterns of relative nutrient consumption as a function of increase in activity. The model was parametrized with data reported in [8], and the transitions between different zones of relative nutrient consumption as a function of increased effort are captured explicitly through incorporation of ventilatory thresholds, particularly VT1 and VT2. Normally, a metabolically healthy individual’s baseline remains below the VT1 threshold, where primary mode of metabolism is fatty acid oxidation. As exercise intensity increases, VT1 is crossed, which is when fatty acid oxidation decreases, and carbohydrate consumption increases. With further increase in exercise intensity, fatty acid oxidation decreases to its minimum and is replaced nearly completely by carbohydrate metabolism, both aerobic and eventually anaerobic, as evidenced by increased levels of blood lactate. These patterns are shown in the data reported in [8] and are captured by the model in Figure 2. I then use this model to simulate projected nutrient consumption profiles for when simulated effort is “stuck” either between VT1 and VT2, or above VT2. I project that when effort is between VT1 and VT2, one is expected to still consume both carbs and fats, but when the simulated effort is maintained indefinitely above VT2, fat consumption decreases completely, and carbohydrates become the primary source of fuel (Figure 3). I propose that it is this second case that more closely describes metabolic alterations that occur in cachexia.

Metabolic patterns such as the second case (over VT2) have indeed been observed in cachectic animals. For instance, Eileen White has recently presented data (7 June 2022, DFCI Cancer and Metabolism 2022 Susan Swerling Lectures), in which autophagy, a normal mechanism of cell degradation that allows orderly recycling of cellular components [26,27], was impaired in mice, which resulted in induction of a cachectic phenotype. Defects in intracellular component recycling resulted in profound alterations of systemic metabolism, showing increased dependency on dedicated nutrient stores causing their depletion, and reduced ability to switch between nutrient sources. The ability to switch between carbohydrate and fat utilization as needed is known as metabolic flexibility [4,28,29]; it has been shown to be significantly impaired in individuals with metabolic syndrome and is likely to be impaired in cancer patients as well.

**Figure 4:**
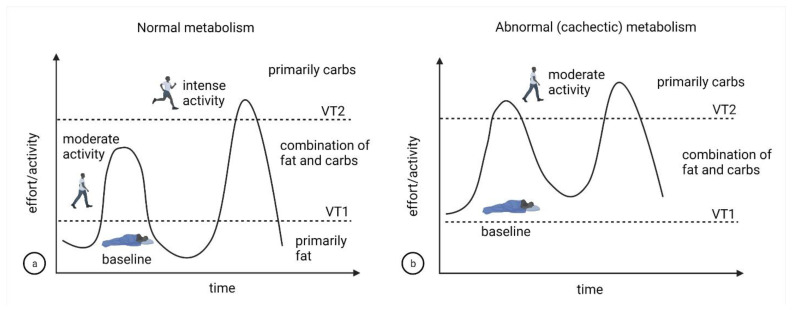
Schematic of proposed differences in metabolism-effort relationships for individuals with (**a**) normal metabolism vs (**b**) cachexia.

In [30], the authors do not explicitly focus on preferential nutrient consumption, but they do report that cachectic mice have elevated levels of lactate (Figures 4C and 6B in [30]), which is consistent with what occurs during prolonged intense exercise at and above the VT2 threshold. In [17], the authors review differences between metabolic adaptations that occur during starvation, protein malnutrition and cachexia, and highlight that paradoxically, basal metabolic rate is elevated in cachexia, which is also consistent with the “metastatic cancer metabolically mimics chronic exercise” hypothesis presented here. A proposed schematic of this hypothesis is shown in Figure 4a,b.

Few real-world examples can be found that could suggest whether extreme chronic exercise can result in cachexia. One possible example, however, could be that of Henry Worsley, who attempted to complete the first unaided solo crossing of the Antarctic, trying to complete the entire 1100-mile continental crossing. According to a description of the trek in Alex Hutchinson’s book “Endure” [31],

“Worsley’s access to near-instantaneous help … allowed him to push much closer to the margins—to empty his tank day after day, after struggling through the snow for 12, 14, or 16 h; to ignore his increasing weakness and 50-pound weight loss…On January 21, his seventieth day of travel, he made the call…The next day, he was picked up for the six-hour flight back to Union Glacier, where logistical support for Antarctic expeditions is based, and then airlifted to the hospital in Punta Aernas, Chile, to be treated for exhaustion and dehydration… In the hospital, though, the situation took an unexpected turn: Worsley was diagnosed with bacterial peritonitis, an infection of the abdominal linings, and rushed into surgery. On January 24th, at the age of fifty-five, Henry Worsley died of widespread organ failure”.

It is possible that the intense ongoing exhaustion that Henry Worsley endured in his exhibition resulted in cachexia, making him more susceptible to infection, having left his body with few resources to fight it and to heal.

## 4. The Obesity Paradox

While it is well known that obesity is associated with increased prevalence of many chronic diseases, including type 2 diabetes, cardiovascular disease and cancer [32,33], in analysis published as early as 1950s [34], if one were to plot the relationship between body mass index (BMI) and longevity, the nadir of the curve would be in the overweight range, resulting in a characteristic “U-shaped” curve. This observation, repeated in multiple meta-analyses of multiple patients, has been termed the “obesity paradox”. A closer look, however, suggests that this may be an artifact of observational data, where very sick individuals often lose weight rapidly, and rapid weight loss is typically accompanied by loss of lean body mass [35,36]. Gonzalez et al. [37], for instance, reported in an observational study of 175 cancer patients whose body composition was assessed before chemotherapy, that the obesity paradox is present only when obesity is defined using BMI; correcting for body composition revealed that obesity predicted higher survival only in the absence of sarcopenia (low muscle mass). That is, it was sarcopenia that was an independent predictor of higher mortality rather than BMI. Daly et al. [38] also reported that loss of skeletal muscle during chemotherapy is prognostic of poor survival in some cancer patients, suggesting that it is body composition rather than BMI that has the protective effect.

Interestingly, Stokes [39] has proposed an approach where, instead of the current BMI, maximum historically attained weight is evaluated with regards to mortality risk, which allows screening for health conditions that may have caused weight loss. The resulting analysis corrects for confounding of illness-induced weight loss, shifting the “optimal” BMI to the leaner range. These results suggest that, while it seems theoretically possible that higher body mass can potentially delay onset of cachexia through greater overall substrate availability, it is more likely that this observation is an artifact of confounding rather than a reflection of an underlying biological mechanism. 

## 5. Validation and Interventions

The hypothesis that was proposed here has circumstantial support described above but requires validation. Given that the methodology for measuring metabolic flexibility has been developed extensively in the area of exercise physiology, many tools can potentially be adapted to measure ventilatory thresholds and metabolic state in patients. For instance, the standard testing protocol described by [8] could be adapted to start collecting measurements at a much lower intensity to assess an individual’s metabolic state. Given the limitations of wearing a mask that covers one’s nose and mouth as needed for indirect calorimetry, a more extensive longitudinal data might be collectable through wearable technologies, such as smart shirts that allow estimating of metabolic thresholds through tracking minute ventilation [11,40], perhaps in combination with breathable devices that allow assessment of respiratory gas exchange [41]. If the proposed hypothesis is validated, wearable technologies may provide a glimpse into potential early warning signs for cancer patients, especially since onset of cachexia is likely to be gradual rather than abrupt, and that in early stages of cachexia changes in metabolic preferences occur before weight loss [42].

Additionally, metabolic thresholds are not static but can be increased through targeted exercise [43]. For instance, increasing the VT1 threshold can for most be achieved through regular brisk walks, as long as they are of sufficient duration; the intensity of threshold-based exercise would increase for fitter individuals, since for professional athletes, for instance, ventilatory thresholds occur at much higher intensity [8]. If the proposed paradigm is validated, this type of training is achievable for most, and might complement additional therapies, such as in the case study reported in [44], where an individualized exercise prescription was used to successfully target oxidative metabolism in a stage IV colorectal metastatic cancer patient. The single case study will clearly need to be replicated in a larger population, but it does suggest possibilities of tailored threshold-based exercise regimens for cancer patients.

## Data Availability

Not applicable.

## Supplementary Materials

The following supporting information can be downloaded at: https://www.mdpi.com/article/10.3390/cells11152317/s1, Table S1: Parameters for Model (1) needed to reproduce Figure 2.
