# Supplementary material for: Understanding Metabolic Alterations in Cancer Cachexia through the Lens of Exercise Physiology
Source: Cells. 2022 Jul 27;11(15):2317. doi: 10.3390/cells11152317 (PMC9367382; doi:10.3390/cells11152317)
Supplement: Supplementary file 1 [file cells-11-02317-s001.zip › cells-1801295-supplementary.pdf]

**Supplementary materials**

for “Understanding metabolic alterations in cancer cachexia through the lens of exercise physiology” by Irina Kareva

*Model description*

The proposed phenomenological model describes the following mechanisms. Effort is introduced extrinsically corresponding to the exercise protocol described in (1), where measurements were recorded after participants warmed up for 15 minutes at an intensity below 100 W. After the warm-up period, participants exercised on a stationary bike, with exercise intensity increasing by 35 W every 10 minutes, until volitional exhaustion. To simulate this level of intensity, Effort is put into the model starting at 100 W at 15 minutes, increasing incrementally to match the reported exercise protocol.

The variables VO2 and VCO2 are described as follows. We assume that when Effort is below threshold VT1, VO2 increases at a rate c1. This is described phenomenologically using the term $c1\frac{(\text{Effort}-\text{VT1})^{-}}{\text{Effort}-\text{VT1}}$, where notation $(\text{Effort-VT1})^{-}$ is positive when Effort < VT1 and is zero otherwise. Similarly, when Effort is below VT1, VCO2 increases at a rate c5. When Effort is greater than VT1 but below VT2, we assume that VO2 increases at a rate c2, which is described using the term $\frac{(\text{Effort}-\text{VT1})^{+}}{\text{Effort}-\text{VT1}}\frac{(\text{Effort}-\text{VT2})^{-}}{\text{Effort}-\text{VT2}}$. Here the term is positive when Effort>VT1 and when Effort is < VT2, and is zero otherwise. Similarly, VCO2 increases at a rate c6. When Effort is greater than VT2 but below VO2max, we assume VO2 increases at a rate c3, and VCO2 increases at a rate c7. Finally, when Effort is greater than VO2max, we assume that VO2 decreases at a rate c4 and VCO2 decreases at a rate c8. Additionally, we introduce parameter kvo2 to describe any potential escape in VO2 and kvco2 to describe any potential escape of VCO2; these parameters may be helpful to phenomenologically allow for the system to return to baseline after Effort is stopped.

The resulting phenomenological system of equations is given below:

$$\begin{aligned}
VO2(t)' &= c1\frac{(\text{Effort}-\text{VT1})^{-}}{\text{Effort}-\text{VT1}} + \text{c2}\frac{(\text{Effort}-\text{VT1})^{+}}{\text{Effort}-\text{VT1}}\frac{(\text{Effort}-\text{VT2})^{-}}{\text{Effort}-\text{VT2}} \\
&\quad + c3\frac{(\text{Effort}-\text{VT2})^{+}}{\text{Effort}-\text{VT2}}\frac{(\text{Effort}-\text{VO2max})^{-}}{\text{Effort}-\text{VO2max}} + c4\frac{(\text{Effort}-\text{VO2max})^{+}}{\text{Effort}-\text{VO2max}} - \text{kvo2 VO2(t)} \\
VCO2(t)' &= c5\frac{(\text{Effort}-\text{VT1})^{-}}{\text{Effort}-\text{VT1}} + \text{c6}\frac{(\text{Effort}-\text{VT1})^{+}}{\text{Effort}-\text{VT1}}\frac{(\text{Effort}-\text{VT2})^{-}}{\text{Effort}-\text{VT2}} \\
&\quad + c7\frac{(\text{Effort}-\text{VT2})^{+}}{\text{Effort}-\text{VT2}}\frac{(\text{Effort}-\text{VO2max})^{-}}{\text{Effort}-\text{VO2max}} + c8\frac{(\text{Effort}-\text{VO2max})^{+}}{\text{Effort}-\text{VO2max}} - \text{kvco2 VCO2(t)}
\end{aligned} \tag{1}$$

Changes in VO2 and VCO2 as a function of exercise intensity can then be calculated at each time point according to equations reported by Frayn (3), such that

$$\begin{aligned} CHOox &= 4.55\ VCO2 - 3.21\ VO2 \\ FATox &= 1.67\ VO2 - 1.67\ VCO2 \end{aligned} \tag{2}$$

where CHOox represents amount of oxidized carbohydrates in g/min, and FATox is the amount of oxidized fat in g/min. Simple arithmetic shows that from available CHOox and FATox data, corresponding respiratory gases can be calculated as

$$\begin{aligned} \mathrm{VO2} &= \frac{1/4.55\ CHOox + 1/1.67\ FATox}{1 - 3.21/4.55} = 0.7463\ CHOox + 2.0332\ FATox \\ VCO2 &= \mathrm{VO2} - 1/1.67\ FATox = 0.7463\ CHOox + 1.4344\ FATox \end{aligned} \tag{3}$$

Table S1. Parameters for Model (1) needed to reproduce Figure 2.

| *Parameter* | **Description** | **PA** | **MA** | **MtS** |
|---|---|---|---|---|
| *c1* | Rate of VO2 increase when Effort < VT1 | 0.12 | 0.11 | 0.1 |
| *c2* | Rate of VO2 increase when VT1 < Effort < VT2 | 0.145 | 0.12 | 0.1175 |
| *c3* | Rate of VO2 increase when VT2 < Effort < VO2max | 0.15 | 0.16 | 0.191 |
| *c4* | Rate of VO2 increase when Effort > VO2max | -0.1 | -0.1 | -0.1 |
| *c5* | Rate of VCO2 increase when Effort < VT1 | 0.152 | 0.1 | 0.05 |
| *c6* | Rate of VCO2 increase when VT1 < Effort < VT2 | 0.207 | 0.118 | 0.116 |
| *c7* | Rate of VCO2 increase when VT2 < Effort < VO2max | 0.25 | 0.1625 | 0.197 |
| *c8* | Rate of VCO2 increase when Effort > VO2max | -0.2 | -0.01 | -0.08 |
| *kvo2* | Clearance of VO2 in the absence of effort | 0.0296 | 0.03 | 0.05 |
| *kvco2* | Clearance of VCO2 in the absence of effort | 0.051 | 0.03 | 0.05 |
| *VT1* | First ventilatory threshold | 220 | 130 | VT1 |
| *VT2* | Second ventilatory threshold | 300 | 170 | VT2 |
| *VO2max* | Threshold of maximum oxygen consumption | 420 | 320 | VT3 |

It should be noted that the full data available for parametrizing this model were not available, particularly for MA and MtS participants, since it was collected at the level of intensity above VT1, and therefore oxygen and carbon dynamics could not be described for any data in that region. The key parameters that qualitatively affected model fits were those describing nutrient dynamics when Effort was above threshold VT1, namely, c2 and c3 for oxygen and c6 and c7 for carbon dioxide, as well as the threshold values VT1, VT2 and VO2max. Even though in this case

values of c1 and c5 could not be estimated except for PA, they are included in the model for use when such data are available. It should also be noted that these are not necessarily unique values of parameters but instead these are the values that allow reproducing with sufficient fidelity data reported in (8), which is sufficient for the purposes of the questions addressed here.
